# Peritoneal Dissemination Complicating Morcellation of Uterine Mesenchymal Neoplasms

**DOI:** 10.1371/journal.pone.0050058

**Published:** 2012-11-26

**Authors:** Michael A. Seidman, Titilope Oduyebo, Michael G. Muto, Christopher P. Crum, Marisa R. Nucci, Bradley J. Quade

**Affiliations:** 1 Division of Women’s and Perinatal Pathology, Department of Pathology, Brigham & Women’s Hospital/Harvard Medical School, Boston, Massachussetts, United States of America; 2 Division of Gynecologic Oncology, Department of Obstetrics and Gynecology, Brigham & Women’s Hospital/Harvard Medical School, Boston, Massachussetts, United States of America; Johns Hopkins Bloomberg School of Public Health, United States of America

## Abstract

**Background:**

Power morcellation has become a common technique for the minimally invasive resection of uterine leiomyomas. This technique is associated with dissemination of cellular material throughout the peritoneum. When morcellated uterine tumors are unexpectedly found to be leiomyosarcomas or tumors with atypical features (atypical leiomyoma, smooth muscle tumor of uncertain malignant potential), there may be significant clinical consequences. This study was undertaken to determine the frequency and clinical consequence of intraperitoneal dissemination of these neoplasms.

**Methodology/Principal Findings:**

From 2005–2010, 1091 instances of uterine morcellation were identified at BWH. Unexpected diagnoses of leiomyoma variants or atypical and malignant smooth muscle tumors occurred in 1.2% of cases using power morcellation for uterine masses clinically presumed to be “fibroids” over this period, including one endometrial stromal sarcoma (ESS), one cellular leiomyoma (CL), six atypical leiomyomas (AL), three smooth muscle tumor of uncertain malignant potential (STUMPs), and one leiomyosarcoma (LMS). The rate of unexpected sarcoma after the laparoscopic morcellation procedure was 0.09%, 9-fold higher than the rate currently quoted to patients during pre-procedure briefing, and this rate may increase over time as diagnostically challenging or under-sampled tumors manifest their biological potential. Furthermore, when examining follow-up laparoscopies, both from in-house and consultation cases, disseminated disease occurred in 64.3% of all tumors (zero of one ESS, one of one CL, zero of one AL, four of four STUMPs, and four of seven LMS). Only disseminated leiomyosarcoma, however, was associated with mortality. Procedures are proposed for pathologic evaluation of morcellation specimens and associated follow-up specimens.

**Conclusions/Significance:**

While additional study is warranted, these data suggest uterine morcellation carries a risk of disseminating unexpected malignancy with apparent associated increase in mortality much higher than appreciated currently.

## Introduction

Uterine leiomyomas (“fibroids”) are common benign uterine neoplasms associated with dysmenorrhea, menorrhagia, pelvic pain and pressure. Surgical procedures commonly employed to treat symptomatic uterine fibroids include myomectomy or sub-total hysterectomy. When performed using minimally invasive techniques, these procedures can be performed on a day surgical basis with limited disability. In order to remove these bulky lesions from the abdominal cavity through laparoscopic ports the tumors must be morcellated [1]. This technique involves fragmenting the lesion such that it can pass through a small incision (i.e. the laparoscope port itself). Originally performed by hand with the assistance of a laparoscopic scalpel, newer methods involve the use of power morcellators, devices designed to draw the lesions into a whirling blade, which then generates small (approximately 1 cm diameter) cores of the lesion, capable of being removed through the port incision. The velocity with which these blades spin has been associated with dispersal of microscopic tumor fragments, thus potentially seeding the peritoneum with small pieces of both neoplastic and non-neoplastic material. This phenomenon is compounded with the fact that some morcellated tumors are not benign [2]. The purpose of this study was to determine the frequency of a post-operative histologic diagnosis of malignancy (leiomyosarcoma), problematic smooth muscle tumors (atypical leiomyoma, smooth muscle tumor of uncertain malignant potential), or variant leiomyomas (e.g. cellular leiomyoma) following power morcellation at a major urban academic medical center, where over 1,000 hysterectomies are performed annually [3]. Additionally, this study determined the frequency of iatrogenic peritoneal dissemination of these lesions secondary to power morcellation and the clinical outcomes data associated with such complications.

**Figure 1:**
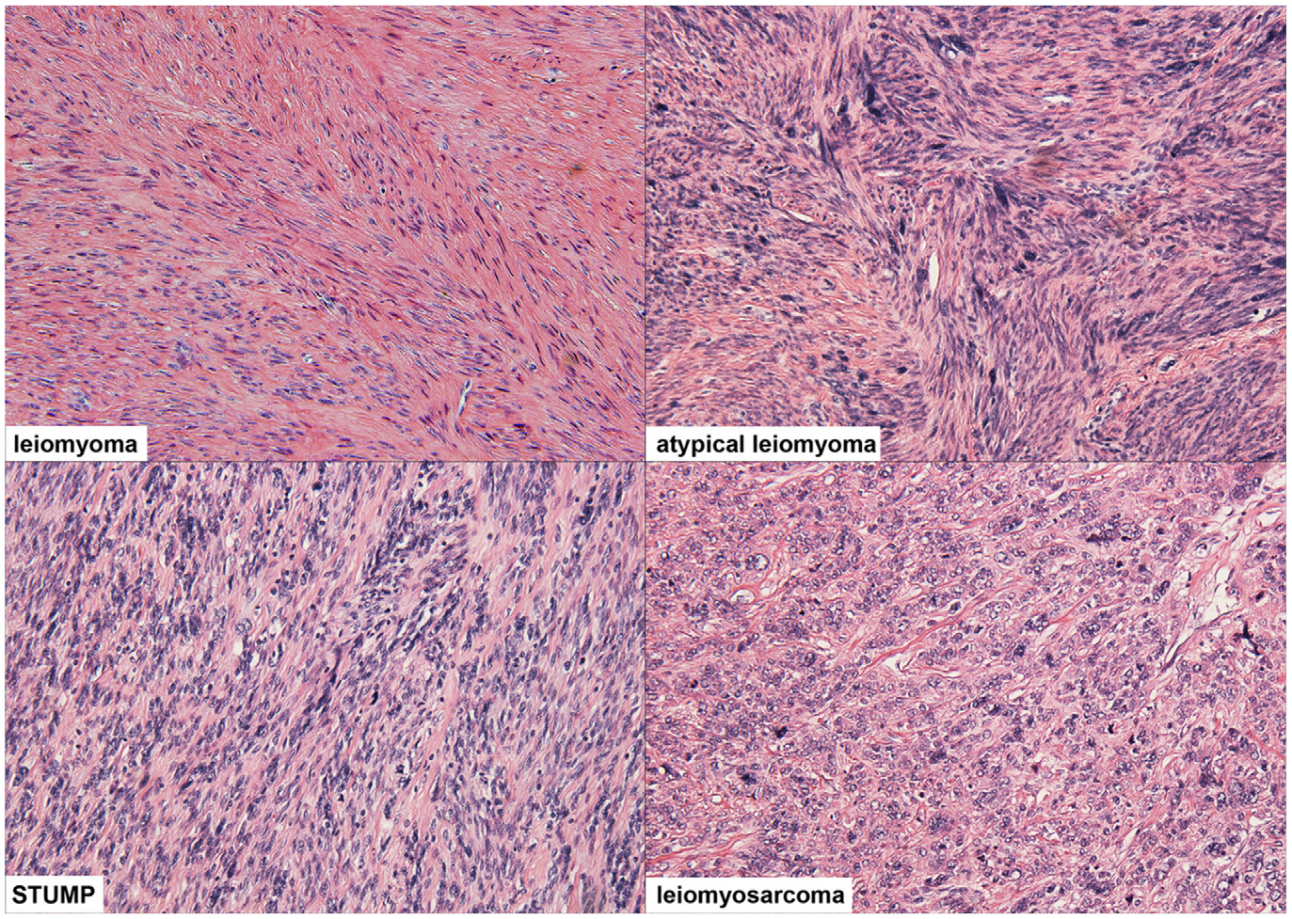
Representative histologic fields of several of the most common diagnoses reviewed during this study. Leiomyoma (case previously published) [6] is characterized by smooth muscle cells without atypia, mitoses, or necrosis. In contrast, atypical leiomyoma (case #7) shows atypia, but rare mitoses (up to 2 mitoses per 10 high power fields). Smooth muscle tumor of uncertain malignant potential (STUMP, case #11) is characterized by a higher rate of mitoses (3 to 8 mitoses per 10 high power fields) and more frequent atypia. Leiomyosarcoma (LMS, case #15) shows significant mitotic activity (over 10 mitoses per 10 high power fields), prominent atypia, and tumor necrosis.

## Methods

### Ethics Statement

All materials from this study were collected in the course of clinical care prior to the start of any research. Research was performed only on discarded tissue, i.e. material deemed not necessary for clinical diagnostics or care. Clinical information used in the study was entirely anonymized from the medical record in accordance with current national guidelines. The Institutional Review Board at Brigham & Women’s Hospital evaluated and approved all aspects of this research, and deemed the procedure eligible for expedited review not requiring subject consent.

### Case Acquisition

The electronic medical records of the Brigham & Women’s Hospital (BWH) Department of Pathology were searched for cases, both in-house and consultation, including the keywords “morcellation” or “morcellated” in association with at least one of the following keywords: myomectomy, fibroid, fibroids, leiomyoma, leiomyomas, leiomyomata, myoma, myomas. Additional cases were identified that contained the keyword “morcellation” or “morcellated” as well as the keyword “uterus”, and that were performed for the indication of uterine leiomyoma, as per the medical record. The search term “leiomyosarcoma” was omitted to avoid case selection bias; however, when this term was included, no additional cases meeting study criteria were identified.

**Figure 2:**
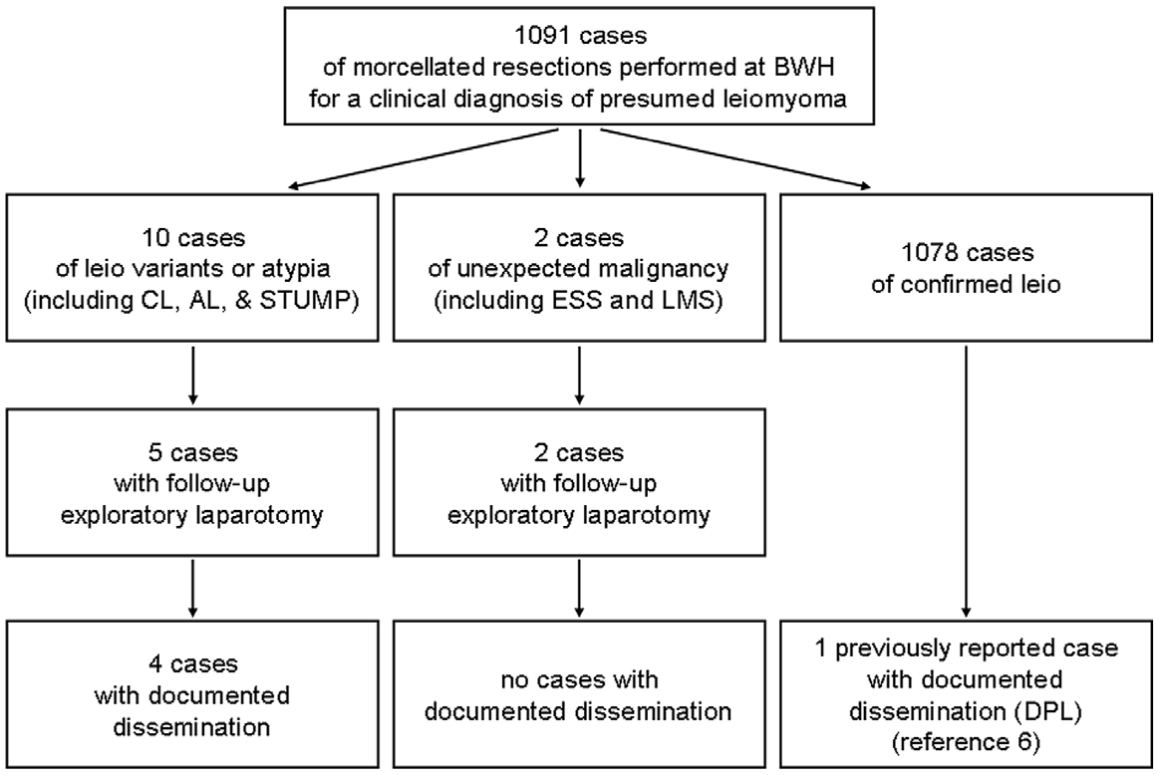
Unexpected diagnosis of leiomyoma variants, atypia, or malignancy following morcellation performed at BWH for clinically presumed uterine leiomyoma.

**Table 1:** Unexpected diagnoses following uterine power morcellation for suspected leiomyoma.

Case #	Age	Diagnosis	Radiologic Size (cm)	Gross Weight (g)	Follow-Up (mos.)
1	48	ESS	8.2	hysterectomy, 322 g	34
2	36	CL	9.7	357	71
3	31	AL	multiple, up to 2.5 cm	382	35
4	35	AL	multiple, up to 8 cm	473	1
5	37	AL	8.0	292	41
6	40	AL	multiple	hysterectomy, 605 g	n/a
7	43	AL	8.0	hysterectomy, 1245 g	6
8	61	AL	multiple, up to 10 cm	hysterectomy, 588 g	32
9	24	STUMP	6.6	120	12
10	41	STUMP	10.0	350	13
11	45	STUMP	multiple, up to 8.7 cm	hysterectomy, 422 g	28
12	42	LMS	multiple, up to 6.2 cm	139	42

Abbreviations – ESS: endometrial stromal sarcoma; CL: cellular leiomyoma; AL: atypical (a.k.a. symplastic) leiomyoma; STUMP: smooth muscle tumor of uncertain malignant potential; LMS: leiomyosarcoma; n/a: not available.

### Specimen Evaluation

Histologic evaluation of morcellated specimens was performed by taking one section of tissue for every 1 cm of the original radiologically reported greatest dimension of the lesion. This was felt to best recapitulate the degree of sampling that would be performed on an equivalent en bloc resection. Diagnoses were rendered as per currently accepted guidelines [4], [5], and included leiomyoma (leio), cellular leiomyoma (CL), atypical leiomyoma (AL), smooth muscle tumor of uncertain malignant potential (STUMP), leiomyosarcoma (LMS), and endometrial stromal sarcoma (ESS). Representative images of cases diagnosed as leiomyoma (case previously published) [6], atypical leiomyoma (case #7), STUMP (case #11), and LMS (case #15) are shown in Figure 1.

**Figure 3:**
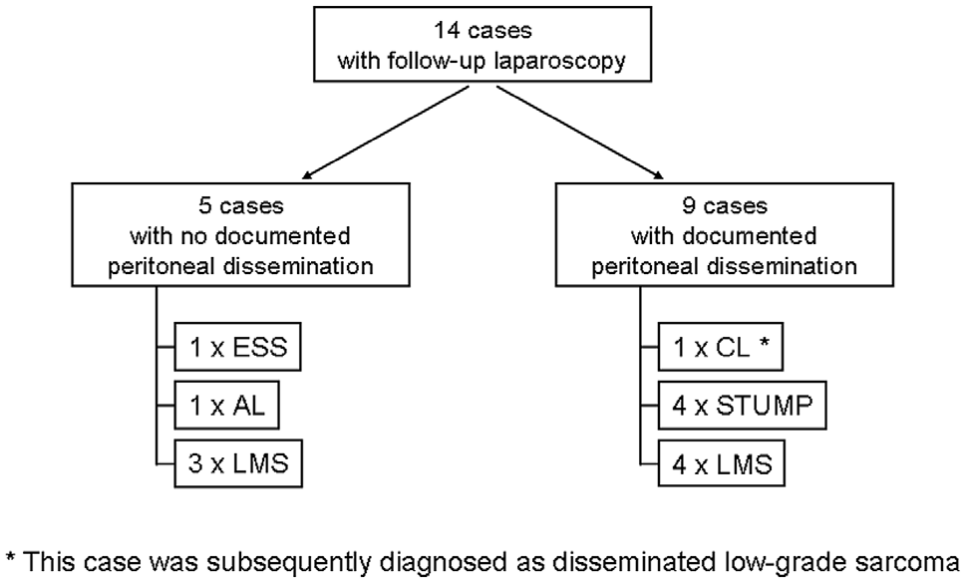
Follow-up exploratory laparoscopy in cases of uterine morcellation with unexpected diagnoses.

**Table 2:** Summary of cases of uterine power morcellation with follow-up exploratory laparotomy.

			First Peritoneal Dx		Subsequent Peritoneal Dx			Clinical Follow-Up		
Case #	Initial Age	Initial Dx	Dx	Interval*	Dx	Interval*	Prolif. Index**	Status	Treatment	Interval*
1	48	ESS	–	1				Alive	none	34
2	36	CL	CL	12	LG sarcoma	18	10%	Alive	arom	71
7	43	AL	–	5				Alive	none	6
9	24	STUMP	STUMP	3			1%	Alive	none	12
10	41	STUMP	STUMP	2				Alive	none	13
11	45	STUMP	STUMP	2			40%	Alive	none	28
13	27	STUMP	STUMP	78				Alive	none	93
12	42	LMS	***	1			5%**	Alive	chemo	42
14	43	LMS	–	1				Alive	none	38
15	47	LMS	LMS	1				Deceased	chemo	17
16	48	LMS	–	1				Alive	none	9
17	49	LMS	LMS	16				Alive	chemo	39
18	58	LMS	Leio	1	LMS	6	80%	Deceased	arom/rads	27
19	68	LMS	LMS	13				Deceased	chemo	29

* Intervals are in months.

** Proliferation indices are measured by MiB-1/Ki-67 staining on the most recently sampled clinical material.

*** While no disseminated disease was identified, residual LMS was identified at the site of the prior hysterectomy. This case is not included in the total number of cases with disseminated disease.

Abbreviations – Dx: diagnosis; ESS: endometrial stromal sarcoma; CL: cellular leiomyoma; AL: atypical (a.k.a. symplastic) leiomyoma; STUMP: smooth muscle tumor of uncertain malignant potential; LMS: leiomyosarcoma; DPL: disseminated peritoneal leiomyomatosis; LG: low grade; arom: aromatase inhibitor therapy; chemo: classic antineoplastic chemotherapy; rads: radiotherapy.

### Statistical Methods

Simple statistics were calculated using the R statistical package. Proportion confidence intervals were calculated using the Wilson method, chosen in light of the small sample sizes in some analyses. Continuous variable confidence intervals were calculated using Student’s *t*-distribution.

**Figure 4:**
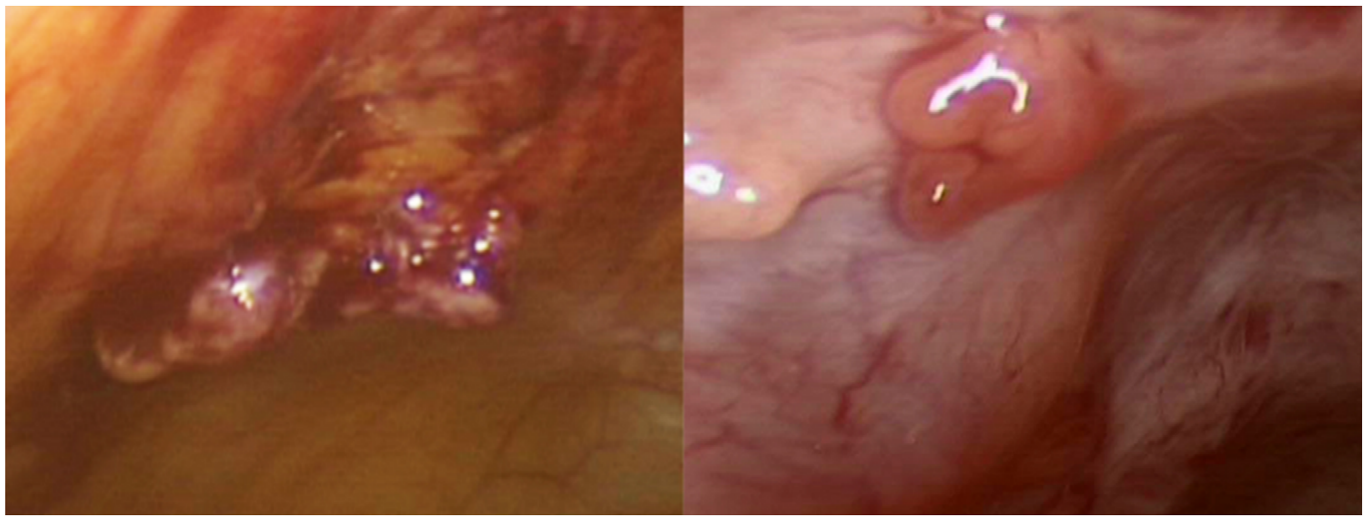
Intraoperative images of nodules on the peritoneal surface, suspicious for disseminated tumor.

**Figure 5:**
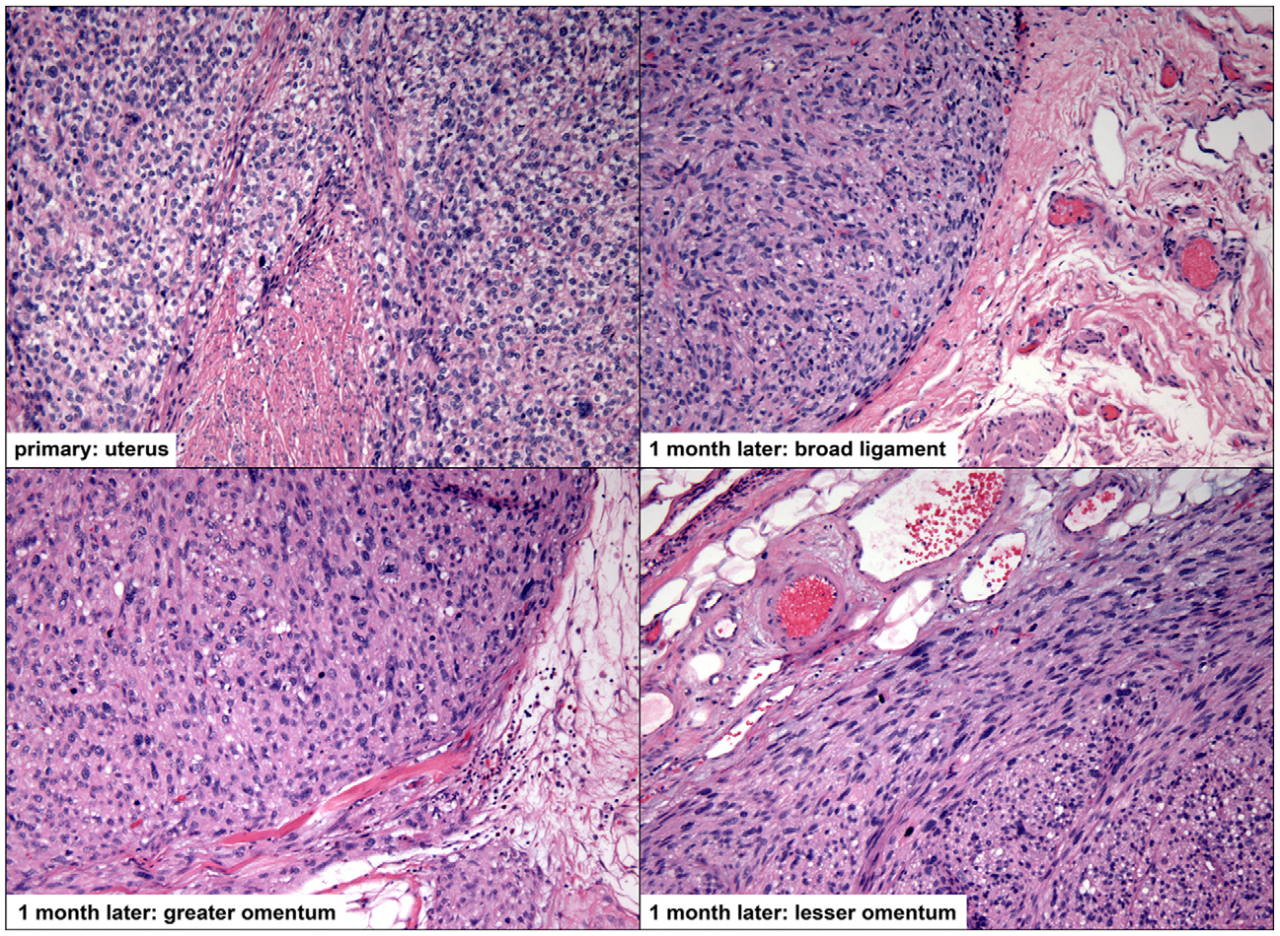
A case of leiomyosarcoma (case #17) diagnosed following a uterine power morcellation, with subsequent diagnosis of dissemination throughout the peritoneum (all images 100x magnification). The primary and disseminated lesions are characterized by very high mitotic rates (all lesions greater than 50 mitoses per 10 high power fields) and significant nuclear atypia and pleomorphism; focal necrosis was also appreciated (not shown).

**Figure 6:**
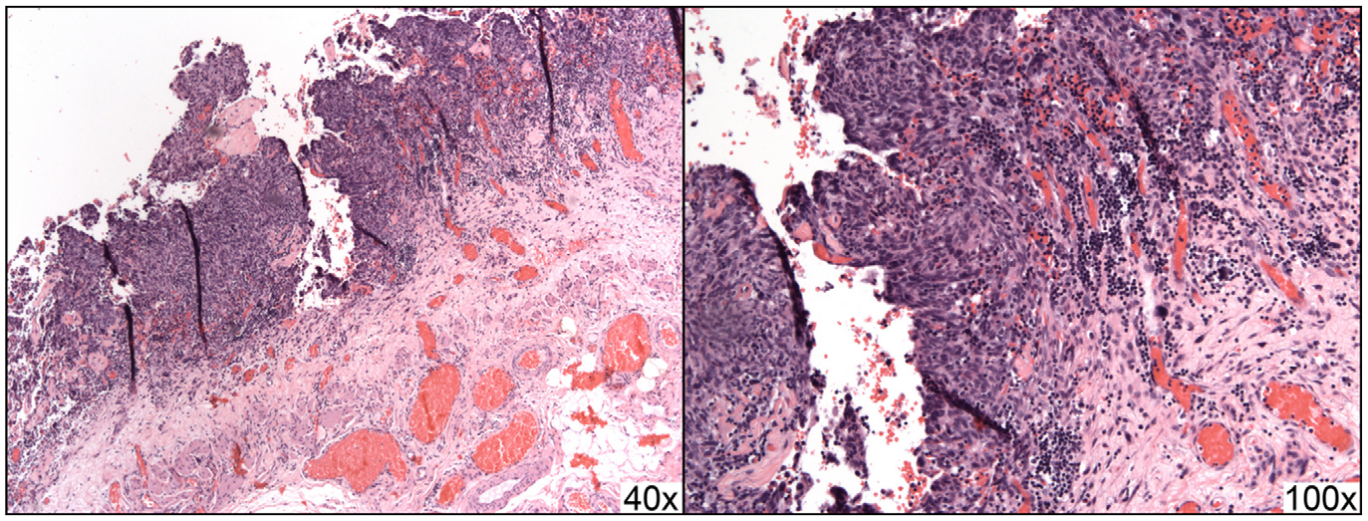
A case of STUMP with peritoneal dissemination (case #11) showing implantation into the omentum. The primary lesion showed scattered marked nuclear atypia and up to 9 mitoses per 10 high power fields; disseminated lesions showed nuclear pleomorphism and atypia as well as increased proliferation indices (40% by MiB-1/Ki-67 staining), but mitoses were not prominent.

## Results

### Unexpected Diagnoses at the Time of Morcellation

From 2005 to 2010, a total of 1091 cases of morcellation were performed at BWH for the indication of uterine leiomyoma. Expert gynecologic pathologists of the Division of Women’s and Perinatal Pathology reviewed tissues from all resections.

Of the 1091 morcellated surgical resections for clinically presumed leiomyoma, thirteen cases were diagnosed as clinically relevant leiomyoma variants, atypical lesions, or malignancy upon histopathologic examination (summarized in Figure 2 and Table 1); all of these cases were found to have been morcellated using a laparoscopic power morcellator. This represents an estimated aggregate incidence of unexpected variants, atypia, and malignancy of 1.2% (95% confidence interval 0.7–2.0%). The ages of patients (range from 36 to 42 years old) did not differ appreciably when grouped by diagnosis. Lesion size was variable, but most of the atypical or malignant lesions were large (over 6 cm); there was a mix of solitary and multiple lesions in all diagnostic categories. Grossly, atypical and malignant lesions occasionally showed yellow coloration, “degenerative” changes, or hemorrhage, but many lesions were not grossly distinguishable from benign leiomyomas.

### Peritoneal Dissemination of Morcellation Lesions

For a subset of cases with unexpected diagnoses at the time of morcellation, follow-up clinical procedures were performed to evaluate for potential iatrogenic peritoneal dissemination subsequent to an unexpected primary diagnosis.

Exploratory laparoscopy was performed in seven of twelve in-house cases described above; original diagnoses in these cases included one ESS, one CL, one AL, three STUMPs, and one LMS. Searching the consult records of BWH for cases where the original morcellation procedure was performed at an outside institution but where tissue from a follow-up laparoscopy was available for review at BWH revealed an additional seven cases of power morcellated uterine mesenchymal lesions originally diagnosed with unexpected atypia or malignancy.

All cases with follow-up, including the seven in-house resections and seven outside hospital resections, were examined to determine if the lesion that had been morcellated could be found disseminated throughout the peritoneum. Of these fourteen cases, nine had documented dissemination (summarized in Figure 3 and Table 2); these data represent an estimated occurrence of morcellator-based dissemination of 64.3% (95% confidence interval 38.8–83.7%). These lesions were grossly visible intraoperatively (Figure 4). Histologically, these lesions were characterized by smooth muscle tissue adjacent to serosal/peritoneal surfaces (Figures 5 and 6).

Disseminated lesions did not show any definite infiltration or invasion of adjacent tissue. Consequently, presence or absence of significant cytologic atypia, mitoses, and necrosis were the basis for determining whether the disseminated tissue represented a benign, atypical, or malignant lesion. In a small number of cases, immunohistochemical staining for smooth muscle actin and/or desmin was used to confirm further that the lesions represented smooth muscle rather than a reactive fibroblastic proliferation.

Proliferation indices (MiB-1/Ki-67 staining) were also evaluated in some of these lesions. Material from a case of iatrogenic disseminated peritoneal leiomyomatosis (DPL) following uterine morcellation showed a proliferation index of 1% [6]. A similar proliferation index was identified in one case of disseminated STUMP (case #9). In contrast, another case of STUMP showed a proliferation index of 40% (case #11). Residual LMS in case #12 showed a proliferative index of 5%. Disseminated sarcoma showed proliferative indices of 10% (case #2) and 80% (case #18).

Follow-up clinical data showed that the only mortality in this case series occurred in patients with diagnoses of leiomyosarcoma; all other patients remain alive at last follow-up. Of the seven LMS cases for which follow-up was available, four showed evidence of peritoneal dissemination (57.1%, 95% confidence internal 25.0–84.2%). Of these, three patients have died (75%, 95% confidence internal 30.1–98.7%), with an average post-diagnosis survival of 24.3 months (95% confidence interval 8.4–40.3 months). The remaining patient with documented dissemination was alive at 39 months, and the cases without dissemination were alive with an average follow-up interval of 29.7 months, including one case (#12) with residual LMS identified at the site of prior hysterectomy on re-exploration but without any evidence of disseminated disease.

## Discussion

Power morcellation, while an effective technique to facilitate minimally invasive surgery of even large uterine masses, carries a risk of dispersing the tumor into the peritoneal cavity. This is particularly concerning considering the inevitable albeit uncommon occurrence of a potentially malignant tumor that is preoperatively misclassified and morcellated on the assumption that it is benign [7]–[9]. The findings presented here add to the existing literature of case reports of both non-neoplastic and neoplastic tissues being disseminated throughout the peritoneum as a result of power morcellation, including tissue of both endometrial (endometrium, endometriosis, endometrial adenocarcinoma, stromal sarcoma) and myometrial (myometrium and the spectrum of smooth muscle neoplasms) origin [6], [10]–[21]. Of note, one of the 1078 cases of uterine morcellations performed at BWH with a post-operative diagnosis of leiomyoma has previously been described as being associated with subsequent development of disseminated peritoneal leiomyomatosis (DPL) [6]. Despite these multiple case reports, no prospective study of these complications has been performed to date.

### Unexpected Diagnoses at the Time of Morcellation

The data in this study show that unexpected diagnoses of variant leiomyoma, atypia, or malignancy will occur in approximately 1% of cases with a preoperative diagnosis of benign uterine leiomyoma. The in-house incidence of unexpected leiomyosarcoma was 0.09% (95% confidence interval 0.00–0.52%). This rate is similar to that reported by Leibsohn *et al*. [2], but much higher than the empiric rate of 1-in-10,000 typically quoted to patients during their pre-procedure briefing.

This estimate, however, may be limited by the assumption that all cases of atypia or malignancy were identified in the original morcellation specimens. One of the cases (#2) studied herein, however, challenges this assumption in that the primary diagnosis was cellular leiomyoma, but the patient later presented with low-grade sarcoma in the peritoneum. It is not possible to know whether this represents transformation after dissemination or if the original specimen contained unsampled sarcoma. At this time, this is the only case in this series in which an unappreciated diagnosis in the original specimen has come to clinical attention, but the actual incidence of unexpected atypia may be higher than the stated estimate.

Of note, this study likely included some morcellations performed by hand rather than with a power morcellator due to the methods of case identification. All cases of unexpected diagnoses were confirmed to have been performed by power morcellation, and as such the actual incidence of unexpected diagnoses associated with this procedure may be slightly higher than the values stated.

### Peritoneal Dissemination of Morcellation Lesions

The data demonstrate that following power morcellation with an unexpected diagnosis of leiomyoma variant, atypia, or malignancy, exploratory laparoscopy will find evidence of peritoneal dissemination 64.3% of the time. Of note, however, mortality has only been ascribed to cases of documented sarcoma with dissemination.

Of the four disseminated sarcoma cases, three patients have died (average survival 24.3 months); one remains alive 39 months following her initial diagnosis. In contrast, the cases of LMS without evidence of dissemination are alive with an average follow-up of 29.7 months. These data are in line with reports by other authors showing that disseminated disease secondary to morcellation increases the mortality of LMS [8], [19], [22], and suggest that iatrogenic implants of LMS behave biologically similarly to metastases.

In the case of disseminated lesions of AL and STUMP, it is not clear at this time if implants have any significant biologic consequences. Additional follow-up is required to determine if cases with peritoneal implants show any different outcomes than those cases without such implants. The current length of follow-up (averaging less than three years) may not be sufficient to identify increased morbidity or mortality for dissemination of such lesions.

Distinguishing reactive changes status post surgery from disseminated low- or intermediate-grade lesions can be challenging because the amount of neoplastic tissue may be limited and the neoplastic tissue may be admixed with fibrosis and chronic inflammation reactive to tumor and/or surgical injury. Recognizing that myofibroblasts show similar immunohistochemical staining profiles to smooth muscle neoplasms, the best method to distinguish neoplasm from reactive changes is comparison of histology from the primary resections and potential disseminated lesions. Case #18 is the only case identified thus far where the first explorative laparoscopy revealed only benign disease but subsequent exploration revealed dissemination of the original malignancy; it is possible that this case represents dissemination of both benign and malignant tissue and that the malignant tissue was not fully appreciated at the first exploratory laparoscopy. This further supports the concern that given additional follow-up time more of these cases will come to clinical attention with disseminated disease.

In view of the challenges in these diagnoses, we recommend the following procedures. In the case of solitary lesions, one section of morcellated tissue should be submitted for histologic evaluation for every 1 cm of the original radiologically reported greatest dimension of the lesion. However, because cases with multiple lesions also carry risk for unexpected diagnoses, we also recommend generously sampling these cases, aiming to cut one section per 1 cm of the dominant lesion(s), as well as several sections representing any secondary lesions. Histologic evaluation should be sure to sample any areas of yellow coloration (as opposed to tan), any softened or “degenerated” areas, tissue adjacent to necrosis, and any areas of hemorrhage, as, with en bloc resections, these findings may correlate with a higher grade (i.e. atypical or malignant) lesion.

For disseminated lesions we recommend comparing histology between the primary tumor and the biopsies taken from throughout the peritoneum. Histologically, the best indicator of dissemination is the presence of bundles of smooth muscle cells involving the peritoneal surface; in this series, infiltration/invasion was not helpful in identifying these lesions, although if present it would strongly suggest dissemination of the neoplastic lesion. It is unclear if MiB-1 proliferation indices help in these cases, given the small sample for which MiB-1 staining was performed in this study; variable intensity and potential sampling issues further limited these stains. The very low rate obtained in at least one histologically malignant lesion (case #12), in particular, raises concerns about the ability of this stain to reliably distinguish a low grade from a high grade lesion. Nevertheless, no low grade lesions showed indices above 10%, suggesting that a significantly elevated MiB-1 proliferation index has a potentially high positive predictive value.

The data presented here demonstrate that uterine lesions believed preoperatively to represent benign leiomyomata may in fact harbor atypical or malignant features at a clinically relevant rate. Furthermore, the data show that the use of power morcellation can be associated with the undesired outcome of disseminating such lesions a high fraction of the time. The histologic evaluation both of the primary and the disseminated specimens is, therefore, of critical importance.

## References

[1] Steiner RA, Wight E, Tadir Y, Haller U (1993) Electrical cutting device for laparoscopic removal of tissue from the abdominal cavity. Obstetrics and Gynecology 81: 471–474. 8437807

[2] Leibsohn S, d’Ablaing G, Mishell DR Jr, Schlaerth JB (1990) Leiomyosarcoma in a series of hysterectomies performed for presumed uterine leiomyomas. American Journal of Obstetrics and Gynecology 162: 968–74; discussion 974–6. 10.1016/0002-9378(90)91298-q 2327466

[3] Jonsdottir GM, Jorgensen S, Cohen SL, Wright KN, Shah NT, et al (2011) Increasing minimally invasive hysterectomy: Effect on cost and complications. Obstetrics and Gynecology 117: 1142–1149. 21508754 10.1097/AOG.0b013e3182166055

[4] Crum CP, Nucci MR, Lee KR (2011) Diagnostic gynecologic and obstetric pathology. Philadelphia, PA: Saunders/Elsevier. 1202 p.

[5] International Agency for Research on Cancer ed, Tavassoli FA, Devilee P (2003) World health organization classification of tumours: Pathology and genetics of tumours of the breast and female genital organs. Lyon: IARC Press. 432 p.

[6] Ordulu Z, Dal Cin P, Chong WW, Choy KW, Lee C, et al (2010) Disseminated peritoneal leiomyomatosis after laparoscopic supracervical hysterectomy with characteristic molecular cytogenetic findings of uterine leiomyoma. Genes, Chromosomes & Cancer 49: 1152–1160. 20842731 10.1002/gcc.20824 PMC2955189

[7] Della Badia C, Karini H (2010) Endometrial stromal sarcoma diagnosed after uterine morcellation in laparoscopic supracervical hysterectomy. Journal of Minimally Invasive Gynecology 17: 791–793. 20955991 10.1016/j.jmig.2010.07.001

[8] Einstein MH, Barakat RR, Chi DS, Sonoda Y, Alektiar KM, et al (2008) Management of uterine malignancy found incidentally after supracervical hysterectomy or uterine morcellation for presumed benign disease. International Journal of Gynecological Cancer: Official Journal of the International Gynecological Cancer Society 18: 1065–1070. 17986239 10.1111/j.1525-1438.2007.01126.x

[9] Hagemann IS, Hagemann AR, LiVolsi VA, Montone KT, Chu CS (2011) Risk of occult malignancy in morcellated hysterectomy: A case series. International Journal of Gynecological Pathology: Official Journal of the International Society of Gynecological Pathologists 30: 476–483. 21804400 10.1097/PGP.0b013e3182107ecf

[10] Sepilian V, Della Badia C (2003) Iatrogenic endometriosis caused by uterine morcellation during a supracervical hysterectomy. Obstetrics and Gynecology 102: 1125–1127. 14607029 10.1016/s0029-7844(03)00683-5

[11] LaCoursiere DY, Kennedy J, Hoffman CP (2005) Retained fragments after total laparoscopic hysterectomy. Journal of Minimally Invasive Gynecology 12: 67–69. 15904602 10.1016/j.jmig.2004.12.021

[12] Paul PG, Koshy AK (2006) Multiple peritoneal parasitic myomas after laparoscopic myomectomy and morcellation. Fertility and Sterility 85: 492–493. 16595233 10.1016/j.fertnstert.2005.10.017

[13] Takeda A, Mori M, Sakai K, Mitsui T, Nakamura H (2007) Parasitic peritoneal leiomyomatosis diagnosed 6 years after laparoscopic myomectomy with electric tissue morcellation: Report of a case and review of the literature. Journal of Minimally Invasive Gynecology 14: 770–775. 17980343 10.1016/j.jmig.2007.07.004

[14] Moon HS, Koo JS, Park SH, Park GS, Choi JG, et al (2008) Parasitic leiomyoma in the abdominal wall after laparoscopic myomectomy. Fertility and Sterility 90: 1201.e1–1201.e2. 10.1016/j.fertnstert.2007.08.068 18410930

[15] Kumar S, Sharma JB, Verma D, Gupta P, Roy KK, et al (2008) Disseminated peritoneal leiomyomatosis: An unusual complication of laparoscopic myomectomy. Archives of Gynecology and Obstetrics 278: 93–95. 18193441 10.1007/s00404-007-0536-9

[16] Miyake T, Enomoto T, Ueda Y, Ikuma K, Morii E, et al (2009) A case of disseminated peritoneal leiomyomatosis developing after laparoscope-assisted myomectomy. Gynecologic and Obstetric Investigation 67: 96–102. 18946223 10.1159/000164949

[17] Epstein JH, Nejat EJ, Tsai T (2009) Parasitic myomas after laparoscopic myomectomy: Case report. Fertility and Sterility 91: 932.e13–932.e14. 10.1016/j.fertnstert.2008.08.014 18922520

[18] Larrain D, Rabischong B, Khoo CK, Botchorishvili R, Canis M, et al (2010) “Iatrogenic” parasitic myomas: Unusual late complication of laparoscopic morcellation procedures. Journal of Minimally Invasive Gynecology 17: 719–724. 20655285 10.1016/j.jmig.2010.05.013

[19] Park JY, Park SK, Kim DY, Kim JH, Kim YM, et al (2011) The impact of tumor morcellation during surgery on the prognosis of patients with apparently early uterine leiomyosarcoma. Gynecologic Oncology 122: 255–259. 21565389 10.1016/j.ygyno.2011.04.021

[20] Cucinella G, Granese R, Calagna G, Somigliana E, Perino A (2011) Parasitic myomas after laparoscopic surgery: An emerging complication in the use of morcellator? Description of four cases. Fertility and Sterility 96: e90–6. 21719004 10.1016/j.fertnstert.2011.05.095

[21] Al-Talib A, Tulandi T (2010) Pathophysiology and possible iatrogenic cause of leiomyomatosis peritonealis disseminata. Gynecologic and Obstetric Investigation 69: 239–244. 20068330 10.1159/000274487

[22] Perri T, Korach J, Sadetzki S, Oberman B, Fridman E, et al (2009) Uterine leiomyosarcoma: Does the primary surgical procedure matter? International Journal of Gynecological Cancer: Official Journal of the International Gynecological Cancer Society 19: 257–260. 19396005 10.1111/IGC.0b013e31819a1f8f

